# Recent Advanced in the Treatment of Advanced SCC Tumors

**DOI:** 10.3390/cancers14030550

**Published:** 2022-01-22

**Authors:** Nicole Basset-Seguin, Eve Maubec

**Affiliations:** 1 Department of Oncodermatology, Université de Paris, 75010 Paris, France; 2 Policlinique de Dermatology, Hôpital Saint Louis, 75010 Paris, France; 3 Department of Dermatology, Hôpital Avicenne, Assistance Publique-Hôpitaux de Paris (APHP), 93000 Bobigny, France; eve.maubec@aphp.fr; 4 Campus de Bobigny, Université Sorbonne Paris Nord, 93017 Bobigny, France

**Keywords:** advanced epidermoid carcinoma, treatment immunotherapy

## Abstract

**Simple Summary:**

Suamous cell carcinoma (SCC) is the most frequent skin carcinoma after basal cell carcinoma. Advanced squamous cell carcinoma (aSCC) are tumors not treatable by surgery or radiotherapy. They represent a rare subgroup of SCC for which no standardized treatment has not been available and chemotherapy was so far mostly palliative. Indeed, their prognosis was very poor. The development of immunotherapy has modified the outcome of such tumors. The rationale for immunotherapy for these tumors is the high mutational burden and their significant increase in incidence in immunosuppressed patients. This review aimed to present the definition of aSCC and discuss the different therapeutical options and treatment modalities.

**Abstract:**

Squamous cell carcinoma (SCC) is the second most frequent form of skin cancer after basal cell carcinoma. While most SCC can be treated by surgery or radiotherapy, some progress into an advanced form and are no longer suitable for these treatments. Guidelines and staging systems have help to define these advanced SCC (aSCC), for which prognosis was very poor until recently. Platin-based chemotherapy was traditionally used, but few prospective trials and no treatment regimen was recommended. Furthermore, toxicity in elderly patients limited its use. The development of immunotherapy has improved the prognosis of these difficult-to-treat aSCC. In this review, we define high risk and aSCC and explored current treatment strategies for these tumors.

## 1. Introduction

Cutaneous epidermoid carcinoma (SCC) are the second most frequent skin cancer after basal cell carcinoma. SCC incidence is rising throughout the world. While most SCC can be treated efficiently with surgery, some progress locally to lymph nodes and eventually lead to distant metastasis. It has been reported that in southern and central USA the death rate from aSCC is comparable to that of melanoma [1].

In this article, we will focus mainly on these high risks and aSCC which are associated with high patient morbidity and mortality. Their prognosis is changing with the development of immune check point inhibitors. It is crucial to identify these high risk patients to choose optimal treatment approaches.

## 2. Definition of High Risk SCC

European guidelines have recently worked to create a definition of high risk factors of recurrence and progression for SCC.

These factors include the following factors: the clinical size, the histopathological subtype and the thickness, the location, the differentiation, the presence of perineural involvement and vascular thrombi, presence of immunosuppression (see Table 1).

The American Joint Committee on cancer (AJCC 8th edition) [2] and the Brigham and Women’s hospital (BWH) [1] proposed slightly different classifications (Table 2 and Table 3). The AJCC8 classification was developed for head and neck SCC and its use might not be relevant to all SCC. The BWH classification system is based on the absence or presence of risk factors (Table 3) The BWH staging system shows overlap with the AJCC-8 both in high-stage and low-stage tumor assignment and allows for the classification of cSCCs beyond the head and neck area.

High risk cSCC correspond to BWH stage T2b/T3 and AJCC stage IV. AJCC stage IV corresponds to a rather heterogenous group of patients some having local involvement while others have locogerional or distant metastasis.

A recent German study reported that among 190 advanced SCC patients, 58% started with a primary low stage tumor (T in situ, T1 and T2), emphasizing the need to assess high risk factors in common SCC [3].

Advanced squamous cell carcinoma (aSCC) are tumors not treatable by surgery or radiotherapy. 

## 3. Lymph Node Involvement in SCC

Lymph node involvement by SCC increases the risk of recurrence and mortality (survival rate: 30% at 5 years), meaning that regular lymph node palpation and ultrasound is highly recommended for high risk tumors [4].

The risk of nodal metastases is 21% to 30% for BWH T2b tumors and 50 to 67% for BWH T3 tumors.

In case of clinical suspicion or ultrasound detection of nodal involvement, fine needle biopsy for histological confirmation is recommended. A positive-node biopsy leads to a lymphadenectomy of the associated nodal basin with or without adjuvant radiation therapy (depending of the number of affected nodes and the presence of extra capsular invasion) [5,6].

In case of lymph node extension, a CT scan or petscan is recommended to search for distant metastasis.

### Is Sentinel Lymp Node (SLN) an Option for High Risk SCC?

As lymph node involvement is a strong prognostic marker for SCC, the potential for earlier detection by SLN biospy has been studied; however no conclusive data on its prognostic information are available.

SLNB efficiency was analysed in a large study (*n* = 847) of SCC cases in the oral cavity and oropharynx, with positive SLNB results observed in 18–60% of patients with high sensitivity (93%) [7,8], but lymph node involvement is much more frequent in these sites compared to cSCC. SLNB was therefore proposed to complete the staging procedure for cSCC. However, the exact impact of SLNBs on cSCC remains unclear and controversial and so far not recommended [5].

A retrospective study evaluating the impact of SLNB was carried out in our department. A total of 37 patients (Saint Louis Hospital, Paris, France), who underwent SLNB, were analyzed together with 290 cases from a systematic review of the literature [9]. The mean rate of positive SLNB was 0.14. In our study, the sentinel lymph node status did not affect relapse-free survival and overall survival (log-rank test; *p* = 0.08 and *p* = 0.31, respectively). This suggests that the procedure is not critical for the management of SCC. The presence of a poorly differentiated tumour was the only risk factor associated with a positive SLNB, as well as with relapse, which may indicate that SLNB could be susceptible for high risk tumors. A study of 143 patients, including 17 patients with the SLND procedure and 24 months of follow up, reported a low sensitivity of SLND as 6 out of 17 SLND progressed to metastatic disease despite a negative SLND. In their study tumor thicknesses of >4 mm and recurrent disease were strongly associated with metastasis [10] Future prospective studies are needed to evaluate the impact of SLNB in cSCC.

## 4. Management of Primary High Risk SCC

All high-risk tumors should be discussed by a multidisciplinary committee.

Surgery of high risk primary SCC is always recommended when feasible with surgical margins of 10 mm [11].

Radiotherapy (RT) can be proposed as a primary treatment when patients are not operable. The association of chemotherapy with RT can also be indicated [11]. RT can also be administered as adjuvant treatment in case of limited excision without the possibility to re-excise, in case of peri neural involvement, N2 or greater nodal disease or recurrent lesions. Furthermore [12], a study revealed a better surgical outcome with adjuvant radiotherapy [13].

## 5. Management of Advanced Cutaneous Squamous Cell Carcinoma

### 5.1. Advanced SCC Had a Very Bad Prognosis before the Development of Immune Check Points Inhibitors (ICI)

A study reported a 6% survival rate for stage IV SCC treated with conventional therapy [14]. Platin-based chemotherapies are usually recommended, but few prospective trials are available. Their overall response rate (ORR) is high, although such results do not last long and its use is limited by toxicity [15]. Few retrospective series and some case reports evaluating stage IVc SCC survival have been published and only a few reports are available concerning the response to chemotherapy and its durability. A study of 36 stage IV cSCC patients [16] reported a 5-year OS of 26% with a median duration of follow up of 22.4 months. Brunner et al. in another study including 603 stage IV head and neck cSCC patients, found [17] a 5-year OS of 11% in the metastatic group, 75% in the «N2M0» group, and 65% in the «N3M0» group (median follow up duration 25.2 months). Hillen et al. reported [5,17] a 3-year overall survival of 26% for their advanced cSCCs cohort. but it included a lower number of severe forms: only 50% of stage IV, a majority of T1–T2, of N0 (73%) only 10% of T4 and only 2% of distant metastatic forms. Similarly, their series included less immunocompromised patients (12%) and patients were given systemic treatment in only 33% of the cases (mostly cetuximab and only three patients were treated with platinum-5FU-cetuximab). This underlines the heterogeneity of stage IV cSCC patients.

### 5.2. EGFR Targeted Therapy

The EGFR family incudes EGFR and human epidermal growth factor receptor (HER)-2, 3 and 4. EGFR is strongly expressed in metastatic cSCCs, and its overexpression in primary cSCCs is a risk factor of poor outcome [18].

Anti-EGFR treatments are monoclonal antibodies, such as cetuximab or panitumumab, competitively inhibiting EGFR, or small molecules, e.g., gefitinib or erlotinib, targeting the intracellular domain of the receptor. Anti-EGFR treatment provided promising results in several trials [19]. A French phase-3 trial prospectively evaluated cetuximab as 1rst-line monotherapy on 36 patients with metastatic (*n* = 3), regional (*n* = 16) or locally advanced (*n* = 17) cSCCs [20]. The ORR was 28%, i (2 CRs and 8 PRs), and the overall disease-control rate was 69% (25/36 patients). Median PFS was 4 months. The median duration of response was 7 months and the mean OS was 8 months. Among the adverse events, the most frequent were infections (22%) and bleeding (11%). An open label study reported the interest of combining platinum salt with cetuximab, thereby allowing for a prolonged PFS [21]. This study suggested that cetuximab could be an option to control the disease in elderly patients for whom chemotherapy is not an option.

However, phase 3 trials are still required to verify the efficacy of anti EGFR in advanced SCC. If anti-EGFR are not approved to treat advanced SCC, cetuximab is listed in the National Comprehensive Cancer Network (NCCN) compendium as a therapy for recurrent and metastatic cSCCs.

The efficacy of small molecules erlotinib and gefitinib seems to be lower with OR of 10 to 32% [22,23].

### 5.3. ICI Have Changed the Prognosis of Advanced SCC

ICI target the PD1 receptor. The PD-1 receptor is expressed on T cells. T cells bind to its ligand (PD-L1) (expressed in 30%–50% of aSCC), thereby inhibiting T-lymphocyte functions [24]. The impact of the immune system on the development of SCC is illustrated by their high incidence in organ transplant recipients (OTR) [25]. Additionally, the high mutational load found in cSCC as well as the frequent presence of tumor infiltrative lymphocytes mean that they are a good candidate for immunotherapy [26]. The PD-1 inhibitor, cemiplimab, was the first approved as a first line agent for locally advanced and metastatic SCC.

In a phase-2 study, cemiplimab (3 mg/kg every 2 weeks) was associated with a response in about 50% of patients enrolled with locally regional or distant metastatic SCC (*n* = 85) and treated for up to 48 weeks. More than half of the patients received systemic treatment before cemiplimab. Median time to response was 2 months; 7% of patient were in complete response. Furthermore, median PFS and OS had not been reached. The median duration of response exceeded 6 months [27]. The estimated proportion of patients with ongoing response at 12 months from the first objective response was 87.8% (95% CI: 78.5% to 93.3%), with the median DOR not reached [28].

Among the potential adverse events, the most frequently observed were fatigue, rash and diarrhea. Some serious immune-mediated and adverse events were reported such as pneumonitis, hepatitis, colitis, adrenal insufficiency, dysthyroidism, diabetes mellitus and/or nephritis, and, unlike other anti-PD-1 inhibitors, infusion reactions. These adverse event led to treatment discontinuation in 7% of patients.

Cemiplimab was approved for patients with metastatic or locally advanced cSCCs who were not candidates for curative surgery or radiation by the US Food and Drug Administration (FDA) in September 2018 and European Medicines Agency (EMA) in July 2019. The recommended cemiplimab dose and schedule is now 350 mg, infused intravenously over 30 min every 3 weeks.

In real life, some of the patients receiving anti-PD1 were immunocompromised, mainly with chronic lymphocytic leukemia cases or other blood disorders [28,29]. Interestingly, in both the two largest Italian and French studies [29], the response rate, PFS and OS were of the same order in immunocompromised or immunocompetent patients, suggesting that it would be of interest to develop specific trials for immunocompromised patients according to types of immunosuppression.

An analysis of real-life data recently identified clinical factors associated with response and survival to cemiplimab. An Italian study including 131 patients found that prior chemotherapy and altered Eastern Cooperative Oncology Group performance status (PS) were associated with progression [29], whereas in a French study that included 240 patients receiving cemiplimab, a multivariate analysis found PS ≥ 2 as being associated during the first 6 months with PFS and OS. However, anti-PD-1 should remain the first line treatment for patients with SCC who have an altered PS, as approximately one third of patients with PS ≥ 2 respond to cemiplimab [29]. Moreover, depending on the studies, head-and-neck location was associated with longer PFS [30] in multivariate analysis in the French study and with a better ORR in univariate analysis in the Italian study [29].

## 6. Pembrolizumab

The efficacy of pembrolizumab was evaluated during several trials. The Keynote 629 was a multicenter, multicohort non randomized, open-label trial. Patients received Pembrolizumab (200 mg IV every 3 weeks) until disease progression, or unacceptable toxicity. The treatment lasted a maximum of 24 months. The ORR was 34% and the median response duration was not reached. The most common AE include fatigue, musculoskeletal pain, decreased appetite, pruritus, diarrhea, nausea, rash, pyrexia, cough, dyspnea, constipation, abdominal pain. Immune-mediated side effects include pneumonitis, colitis, hepatitis, endocrinopathies, nephritis and skin adverse reactions [31,32]. A higher ORR of 50% in patients with locally advanced SCC compared to a 35% ORR in patients with recurrent/metastatic was recently demonstrated in an update of the Keynote 629 study. Moreover, the durability of responses was confirmed [33].

The CARSKIN study evaluated first line pembrolizumab in a multicenter open-label trial including a 39-patient primary cohort and an 18-patient expansion cohort. ORR at week 15 was 41% in the primary cohort, and 42% in the overall population. It was significantly higher in PD-L1 positive (55%) than in PD-L1 negative (17%) patients. Only 2/16 responders had PD-L1 negative tumors. At a median follow-up of 22.4 months, the primary cohort’s median PFS, DOR, and OS were, respectively 6.7 months, not reached, and 25.3 months. The most common AE were fatigue, diarrhea, hypothyroidism, pruritus, and eczematous eruption. A death occurred due to fast SCC progression and another unexpected death occurred related to a second aggressive head and neck SCC [34].

Pembrolizumab was approved in June 2020 by the FDA for recurrent and metastatic SCC not curable by surgery or radiation.

Nivolumab efficacy was reported in head and neck tumors [35] and more recently in aSCC (NCT03834233).

A recent meta-analysis including 7 trials evaluating anti PD-1 in advanced SCC with a subgroup analysis of the odds ratio for ORR by PD-L1 tumor proportion score found an OR of 2.81 for ORR in patients with a PD-L1 tumor proportion score of ≥1% [31,34,36,37]. However, patients with PD-L1 negative tumors can sometimes be responders to anti PD-1 and thus other predictive factors are required.

Late complete responders have been reported in all recent trials with anti PD-1 in SCC. A recent publication showed that 9/11 PR patients by RECIST criteria in a cohort of patients receiving immunotherapy for at least 10 months were CR patients by PET [38]. Although these data are yet to be confirmed, this study suggests that PET CT might be preferentially used for anti PD-1 efficacy assessment.

Adjuvant treatment with both cemiplimab (NCT03969004) and pembrolizumab after surgery and radiotherapy in high risk (NCT03833167) are being investigated.

The neoadjuvant use of immune check point inhibitors is also being investigated for both cemiplimab (NCT04154943), pembrolizumab (NCT04808999) and nivolumab (NCT04620200). In a trial of the MD Anderson Cancer Center, 20 patients received neoadjuvant cemiplimab for stage III or IVA SCC of head and neck. Although only 6 patients (30%) had PR by RECIST, 14 patients (70%) had either a pathological CR or a major pathological response illustrating underestimation of pathological response by imaging. At a median follow-up of 23 months, one patient progressed and died, one died without disease, and two developed recurrence [39]. A larger phase 2 trial is ongoing (NCT04154943).

## 7. Follow Up

Patients with aSCC must be followed every 3–4 months with a lymph node ultrasonography, MRI and CT scan.

## 8. Conclusions

Advanced SCC had a poor prognosis. The development of immune check-point inhibitors has changed the overall survival of patients with advanced SCC with prolonged remission. Research is still required to improve these results and identify early markers of progression to optimize treatment strategy.

## Figures and Tables

**Table 1:** Prognosis risk factors for primary SCC (adapted from Stratigos et al., Refs. [2,3]).

Prognosis Group	Tumour Diameter	Location	Depth/Level of Invasion	Histological Features	Surgical Margins	Immune Status
Low risk	Less than 2 cm	Sun exposed (except lip/ear)	Less than 6 mm Invasion above subcutaneous fat	Well-differentiated common variant or verrucous	Clear	Immuno-competent
High risk	More than 2 cm	Ear/lip Non sun expposed sites (sole foot) SCC arising in radiation sites, scars, burns, chronic inflammatory conditions Recurrent SCC	More than 6 mm Invasion beyond subcutaneous fat	Moderately or poorly differentiated grade Acantholytic, spindle, or desmoplastic subtypes	Incomplete excision	Immuno-suppressed Organ transplant recipient Chronic Immunosuppressive disease or treatment

**Table 2:** AJCC 8th edition.

Tx: Primary tumor not accessible
T0: No primary tumor
Tis: in situ Carcinoma
T1: Tumor diameter ≤2 cm
T2: Tumor diameter >2 cm but ≤4 cm
T3 Tumor diameter >4 cm, minor bone invasion, perineural invasion or deep invasion

Perineural invasion is defined as tumor cells within a nerve sheath lying deeper below the dermis, ≥0.1 mm in caliber, with clinical or radiographic involvement of named nerves without skull base invasion or transgression. Deep invasion is defined as that going beyond the subcutaneous fat or >6 mm. T: tumor.

**Table 3:** BHW.

T1: 0 risk factor
T2a: 1 risk factor
T2b: 2–3 risk factors
T3: ≥4 risk factors or bone invasion

Risk factors: Tumor diameter ≥2 cm; Tumor invasion beyond subcutaneous fat (excluding bone invasion, which automatically upgrades to T3); Perineural invasion ≥0.1 mm; Poorly differentiated.
